# Lines Written in Winchester Hospital

**Published:** 1888-06-23

**Authors:** 


### The Old and the New.

### Lines Written in Winchester Hospital.

[In 1444 Cardinal Beaufort appointed, in addition to the first es'ablishment at St. Cross Hospital, two priests, thirty five brethren; and three sisters who were to act as nurses to the sick and infirm. This is the first record of sick nursing in Winchester.]

### THE NEW SISTER TO THE OLD.

From our high-mounted hospital, the goal of every breeze,
I look down on your ancient home among the budding trees,
The pleasant place that lieth grey on the banks of Itchen slow;
Now tell me what was nursing like four hundred years ago?

Were your men always respectful, or did they sometimes swear?
Did you ever long for "forty winks," and know you didn't dare?
Did your women have hysterics, did they quarrel with the food?
Were your small boys ever naughty, were your babies ever good?

Ah no; you had no children, no women—only men;
In truth a world of trouble you were spared, my sister, then;
A world of trouble truly, but a world of pleasure too,
For nothing brightens up the ward as the flowers and babies do.

Did you partake of friendly cups when the day dawned grey at three?
Sweet at all times and seasons, oh! sweetest then is tea;
That you had none whatever, I can't, I won't believe,
A nurse without a tea-pot I hardly can conceive.

Did your aprons get all oily, did your sleeves at elbows wear?
Did your caps grow limp and languid, did your gowns at "gathers" tear?
And did you never venture in the outside world at all?
Were you not rather tired of life, behind your sheltering wall?

Now through the daybreak vapours, I see your shadowy smile.
For you these trivial joys and cares are passed this long, long while,
But your answer to my questioning thoughts, the dewy dawn-wind bore
Across the stream, across the fields, across the centuries four.

### THE OLD SISTER TO THE NEW.

Though different our surroundings, child, yet much the same our lot—
Fashions and modes may alter, human nature changes not;
We began enthusiastic, *that* soon vanished like the dew,
Days seemed endless, life laborious, unto us as well as you.

We had nursing, much devotion, simples, too, to plant and cull,
Lives than yours perhaps more peaceful, lives assuredly more dull;
And we often failed in pleasing, spite of effort and goodwill—
We down in the St. Cross Valley, just like you on Cromwell's Hill.

And our patients? Yes, they grumbled, for the poor will aye complain;
Hard, hard their life in our rude times, hard still doth it remain;
But, child, remember all their stints, sharp cold, and labour hard,
And think not of a few shrewd turns in tempers want hath marred.

The kettles are boiling, the lav'rock sings,
Over the roadway the gaol bell rings;
Heralds these of the opening day.
Fancies and phantoms all vanish away,
Go back to your dreaming, ye ghosts of the night;
Light comes with morning, and labour with light.

